# APP mouse models for Alzheimer's disease preclinical studies

**DOI:** 10.15252/embj.201797397

**Published:** 2017-08-02

**Authors:** Hiroki Sasaguri, Per Nilsson, Shoko Hashimoto, Kenichi Nagata, Takashi Saito, Bart De Strooper, John Hardy, Robert Vassar, Bengt Winblad, Takaomi C Saido

**Affiliations:** ^1^ Laboratory for Proteolytic Neuroscience RIKEN Brain Science Institute Wako Japan; ^2^ Department of Neurology and Neurological Science Graduate School of Medicine Tokyo Medical and Dental University Tokyo Japan; ^3^ Division of Neurogeriatrics Department of Neurobiology, Care Sciences and Society Center for Alzheimer Research Karolinska Institutet Huddinge Sweden; ^4^ Department of Neuroscience and Pathobiology Research Institute of Environmental Medicine Nagoya University Nagoya Japan; ^5^ Dementia Research Institute University College London London UK; ^6^ Department for Neurosciences KU Leuven Leuven Belgium; ^7^ VIB Center for Brain and Disease Research Leuven Belgium; ^8^ Reta Lila Research Laboratories and the Department of Molecular Neuroscience University College London Institute of Neurology London UK; ^9^ Department of Cell and Molecular Biology Feinberg School of Medicine Northwestern University Chicago IL USA

**Keywords:** Alzheimer's disease, amyloid precursor protein, amyloid β peptide, *App* knock‐in, APP transgenic, Molecular Biology of Disease, Neuroscience

## Abstract

Animal models of human diseases that accurately recapitulate clinical pathology are indispensable for understanding molecular mechanisms and advancing preclinical studies. The Alzheimer's disease (AD) research community has historically used first‐generation transgenic (Tg) mouse models that overexpress proteins linked to familial AD (FAD), mutant amyloid precursor protein (APP), or APP and presenilin (PS). These mice exhibit AD pathology, but the overexpression paradigm may cause additional phenotypes unrelated to AD. Second‐generation mouse models contain humanized sequences and clinical mutations in the endogenous mouse *App* gene. These mice show Aβ accumulation without phenotypes related to overexpression but are not yet a clinical recapitulation of human AD. In this review, we evaluate different APP mouse models of AD, and review recent studies using the second‐generation mice. We advise AD researchers to consider the comparative strengths and limitations of each model against the scientific and therapeutic goal of a prospective preclinical study.

## Modeling preclinical AD with APP mice

Alzheimer's disease (AD) is the most common neurodegenerative disease. In 2015, of the ~47 million individuals with dementia worldwide (Prince *et al*, 2016), AD accounted for 50–70% of these cases (Winblad *et al*, 2016). In the coming decades, global AD prevalence is projected to reach higher epidemic levels that will place a massive economic burden on society. Given the grim epidemiological forecast, scientifically based strategies to prevent AD are urgently needed, as evidenced by the fact that in the previous two decades more than 400 medication candidates failed to reach the clinic (Mangialasche *et al*, 2010). This large‐scale failure by the pharmaceutical industry and biomedical research community can be attributed to many factors, including inappropriate choice of mouse models, wrong timing for therapeutic interventions, over‐reliance on inappropriate assays for translational studies, or lack of precise biomarkers, most of which center on efficacy and reproducibility in preclinical studies of which animal models were a critical component. In this review, we focus on the important roles that genetically engineered mouse models have contributed to AD mechanistic studies and preclinical drug development. Recently, new mouse models have been introduced to the community, and we will summarize the construction, characteristics, and merits and demerits of current AD mouse models to facilitate understanding of these tools for the design of future studies. In this aim, we will be guided by the principle that AD is a disease that should ideally be prevented in preclinical stages where a potential interventional window of at least 20 years exists before dementia clinically manifests (Funato *et al*, 1998; Bateman *et al*, 2012).

**Figure 1:**
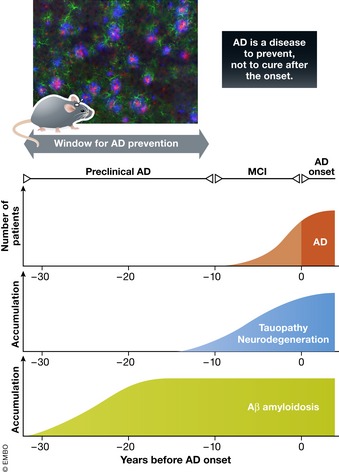
Cortical pathology, neurological symptoms, and APP mouse models of AD There are three neurological phases leading to the onset of AD and associated cortical pathology. The first phase is preclinical AD, where Aβ accumulates in cortex without neurological symptoms. The second phase is mild cognitive impairment (MCI), where tauopathy and neurodegeneration proceed with predementia symptoms. The third phase is AD, where neurodegeneration eliminates neurons and neuronal circuits in an irreversible manner with progressively serious symptoms of dementia. As models of preclinical AD, APP‐overexpressing mice or *App* knock‐in mice exhibit extensive Aβ pathology without tauopathy and neurodegeneration, for which there is a preventive window of approximately two decades. Modified from Ihara and Arai (2007). The pathology shown is from the cortex of a 9‐month‐old *App* ^*NL‐G‐F/NL‐G‐F*^ mouse. Blue: Aβ; red: microglia (Iba‐1); green: astrocyte (GFAP) (Saito *et al*, 2014).

Clinically, AD is characterized by early memory deficits followed by a decline in other cognitive functions (Scheltens *et al*, 2016; Winblad *et al*, 2016). The pathology of AD begins before overt cognitive symptoms and includes the accumulation of amyloid β peptide (Aβ) as extracellular plaques, hyper‐phosphorylated tau as intracellular neurofibrillary tangles (NFTs), and chronic neuroinflammation, followed by the loss of neuronal cells mainly in the cerebral cortex and hippocampus (Braak & Braak, 1991; Hyman *et al*, 2012). In parallel, a coordinated breakdown in vascular, astroglial, and oligodendrocytic responses demonstrates that AD is a systems disorder and the roles and interactions of different cell types in the decline of brain homeostasis and resultant dementia is a major research topic (De Strooper & Karran, 2016). Aβ pathology is initiated at least two decades before cortical tau pathology and the onset of clinical symptoms (Bateman *et al*, 2012; Maruyama *et al*, 2013). After disease onset, it is increasingly difficult to treat symptoms after postmitotic neurons start to degenerate (Fig 1) and finely tuned neuronal circuits and cognitive skills are not easily recovered at later stages. Thus, the development of accurate preclinical animal models of AD for studies of disease mechanisms and the development of medications for early prevention and treatment are considered vital research goals in accord with the global epidemiological status.

Sporadic late‐onset AD (LOAD) accounts for more than 99% of all cases (Campion *et al*, 1999), and the ratio of LOAD patients to all AD patients continues to increase because aging is a primary risk factor aligned with aging of the world population. Early‐onset AD (EOAD), in contrast, is predominately familial and caused by mutations in genes that encode amyloid precursor protein (*App*), presenilin‐1 (*PSEN1* or PS1) and presenilin‐2 (PS2). Proteolytic processing of APP by β‐secretase (β‐site APP cleaving enzyme 1 or BACE1) and γ‐secretase generates soluble Aβ fragments. γ‐Secretase is a protein complex composed of PS1 or PS2, nicastrin, Aph1 and presenilin enhancer 2 (*PEN2*). Most familial AD (FAD) mutations affect processivity of γ‐secretase resulting in the release of longer Aβ peptides and a shift in the relative ratios of the different peptides, including the Aβ_42_/Aβ_40_ ratio (Welander *et al*, 2009; Chávez‐Gutiérrez *et al*, 2012). Similarly, mutations in the *App* gene result in the production of longer Aβ peptides that aggregate more easily (Rosenberg *et al*, 2016). Interestingly, an *App* ^*A673T*^ mutation was claimed to reduce the risk of sporadic AD (SAD) and age‐related cognitive decline by decreasing the production of Aβ (Jonsson *et al*, 2012), although these findings require confirmation (Wang *et al*, 2015) and the mutation appears to affect the biophysical properties of Aβ peptides (Benilova *et al*, 2014; Maloney *et al*, 2014). The existence of familial mutations that directly affect Aβ production and influence AD risk is often cited as evidence that Aβ accumulation is central to AD pathogenesis (amyloid cascade hypothesis: Selkoe & Hardy, 2016).

**Figure 2:**
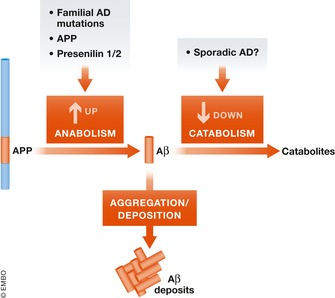
Aβ proteostasis determined by the balance of production and degradation The balance of anabolism and catabolism determines the steady‐state quantity of a given protein in a biological system. In FAD, increased anabolism of pathogenic Aβ (Aβ_42_ and Aβ_43_) in cortex results in pathological deposition. In SAD, the causes of Aβ accumulation are not fully understood, but an aging‐associated decrease in catabolism is a candidate mechanism (Saido & Iwata, 2006; Hellström‐Lindahl *et al*, 2008).

In general, Aβ and tau pathology in sporadic and familial cases are morphologically similar, rationalizing the use of mouse models with genetically engineered FAD mutations for understanding SAD. However, the extent to which these models actually reproduce SAD remains unknown. A critical factor to consider in developing and using mouse APP models is the potential mechanism of Aβ accumulation. In FAD, Aβ deposition is primarily caused by the increased production of Aβ_>40_ except for intra‐Aβ sequence mutations that alter its structural properties (Selkoe & Hardy, 2016) or the Swedish mutation that increases all Aβ species by increasing cleavage at the β‐site. Whether the Iceland mutation *App* ^*A673T*^ is protective by decreasing Aβ production (Jonsson *et al*, 2012) remains controversial. However, because Aβ degradation declines with aging potentially due to a decrease in the major Aβ‐degrading enzyme neprilysin (Iwata *et al*, 2001, 2002; Hellström‐Lindahl *et al*, 2008) and because Aβ clearance is decreased in SAD patients (Mawuenyega *et al*, 2010), Aβ deposition in SAD is likely partially caused by an aging‐associated decrease in degradation/clearance of Aβ (Fig 2). In accord, increased production of Aβ or its decreased degradation/clearance might contribute to Aβ accumulation in AD (Saido & Iwata, 2006). Recent studies on the ubiquitin‐proteasome system and autophagy (Nilsson *et al*, 2013; Ciechanover & Kwon, 2015; Khaminets *et al*, 2016) point to the essential significance of protein degradation in many diseases including AD. While decreased Aβ degradation may be dominant in SAD, most APP mouse models have increased FAD‐like production. The selection of a mouse model for preclinical studies should consider this issue.

Various AD mouse models (Onos *et al*, 2015; Puzzo *et al*, 2015; Drummond & Wisniewski, 2017) as well as other animal models including rats, non‐human primates, *Drosophila*, and *Caenorhabditis elegans* (Drummond & Wisniewski, 2017) have been recently reviewed. In this review, we will focus on genetically modified APP mouse models of AD as they are the most practical approach for *in vivo* screening and validation of preventive medications at this time (Zahs & Ashe, 2010). In the absence of gene manipulations, no small animal models exist at present that sufficiently or consistently mimic clinical disease pathology for experimental and preclinical studies of AD. Furthermore, we will focus on the preclinical stage when the time window for effective prevention and treatment is wider. Preclinical AD patients are cognitively normal (Bateman *et al*, 2012), and likewise, model mice in parallel preclinical stages having Aβ pathology without tauopathy and neurodegeneration should not exhibit strong cognitive impairment. However, at this early stage, pathological alterations in Aβ or tau are thought to initiate disease processes including synaptic dysfunction, local damage to spines and dendrites, and vascular pathology that are observed in AD mouse models and presumably in presymptomatic humans (Ashe & Zahs, 2010). There are other potential points for prevention at this stage, such as mechanism(s) by which Aβ amyloidosis affects tauopathy or by which TREM2 (Guerreiro *et al*, 2013; Jonsson & Stefansson, 2013) influences pathogenesis and these may also be studied in mouse models for Aβ pathology.

## First‐generation mouse models

First, a note on terminology: The term “transgenic (Tg) mice” could be confusing because in a wide sense it means “genetically modified mice” and because it also means mice into the host genome of which transgene is inserted in single or multicopy number. We use the second definition for the Tg mice in this review because knock‐in and knockout mice are different from Tg mice in that they maintain the original murine genomic structure except for the introduced mutations.

Several groups generated Tg mice that overexpress APP with or without FAD mutations using various promoters (Table 1), such as platelet‐derived growth factor‐β (PDGF‐β), prion protein (PrP), and Thy1. Frequently used models include PDAPP (Games *et al*, 1995), Tg2576 (Hsiao *et al*, 1996), APP23 (Sturchler‐Pierrat *et al*, 1997), J20 (Mucke *et al*, 2000), and TgCRND8 (Chishti *et al*, 2001). The APP constructs differ among the lines: They include APP695, APP770, and minigenes. Some mice carry more than one mutation in the transgene, and the most commonly used mutation is the Swedish mutation (K670N/M671L; Citron *et al*, 1992), which causes the overproduction of total Aβ from APP. These mice exhibit extracellular Aβ deposits in the brain, which are reminiscent of plaques in human patients with some differences (refer to section “Limitations of first‐generation mouse models”). In addition, these mice develop cognitive dysfunction before the appearance of amyloid plaques in many cases. However, they are unable to recapitulate neurofibrillary tangle (NFT) formation or neuronal loss.

**Table 1:** Comparison of current APP mouse models of AD

	Strain(s)	Genetic background	Promoter	Mutation(s)	General features	Potential disadvantages	Suitable applications
Single transgenic APP‐Tg	PDAPP	C57B6 × DBA2	PDGF‐β	APP^V717F^	Moderate behavioral phenotype Neuronal loss in some models	Random integration of a transgene Overexpression‐related artifacts: multiple APP fragments overproduced No perfect negative control mice Artificial expression pattern controlled by exogenous promoters No NFTs, a feature of preclinical AD Sudden death (unknown reason): Tg2576, APP23 Cognitive impairment often preceding Aβ accumulation Mixed genetic backgrounds in some cases	Analysis of Aβ production, deposition, and Aβ‐associated neuroinflammation Drug development (targeting Aβ deposits and secretases) Analysis of behavior if caused by Aβ Identification of CSF biomarkers
	Tg2576	B6; SJL mixed background	hamster prion protein (PrP)	APP^KM670/671NL^			
	APP23	C57BL/6	mouse Thy1	APP^KM670/671NL^			
	J20	C57BL/6	PDGF‐β	APP^KM670/671NL,V717F^			
	TgCRND8	Hybrid C3H/He‐C57BL/6	hamster prion protein (PrP)	APP^KM670/671NL,V717F^			
Double transgenic APP‐Tg × PSEN1‐Tg or KI	APPPS1	C57BL/6J	mouse Thy1 (APP, PS1)	APP^KM670/671NL^ PS1^I166P^	Moderate behavioral phenotype Aβ accumulation from early stage Neuronal loss in some models	Random integration of a number of transgenes in some cases Overexpression‐related artifacts: multiple APP fragments overproduced No perfect negative control mice Artificial expression pattern controlled by exogenous promoters Multiple mutations (APP + PS1) Non‐specific ER stress may arise No NFTs, a feature of preclinical AD Mixed backgrounds in some cases Complicated crossbreeding in some cases	Analysis of Aβ production, deposition, and Aβ‐associated neuroinflammation Drug development (targeting Aβ deposits and secretases) Analysis of behavior if caused by Aβ Identification of CSF biomarkers Analysis of cell death, in some cases
	5XFAD	(C57BL/6 × SJL)F1 and C57BL/6J	mouse Thy1.2 (APP, PS1)	APP^KM670/671NL,I716V,V717I^ PS1^M146L,L286V^			
Triple transgenic	3xTg‐AD	C57BL/6J	mouse Thy1.2 (APP, Tau) endogenous (PS1)	APP^K670N,M671L^ PS1^M146V^ MAPT^P301L^	Moderate to severe behavioral phenotype NFT formation Neuronal loss	Random integration of transgenes Overexpression‐related artifacts: multiple APP fragments overproduced No perfect negative control mice Artificial expression pattern controlled by exogenous promoters Multiple mutations (APP + PS1 + FTDP‐17) where FTDP‐17 mutations are not causes of AD Mixed genetic backgrounds Complicated crossbreeding Cognitive impairment preceding Aβ accumulation	Analysis of Aβ production, deposition, and Aβ‐associated neuroinflammation Drug development (targeting Aβ and tau) Analysis of behavior if caused by Aβ and tau Tau imaging Identification of CSF biomarkers Analysis of cell death
Single App knock‐in	NL‐F	C57BL/6J	endogenous APP	APP^KM670/671NL,I716F^	Minor behavioral phenotype No overexpression of APP and byproducts except for CTFβ Endogenous *App* promoter‐driven gene expression Presence of relevant control mice (NL mice) Two lines for differential purposes NL‐F (wild‐type Aβ) NL‐G‐F (Arctic Aβ) : Aβ accumulation from early stage	Multiple familial AD mutations of APP, the interaction between which has not been identified Unknown effects of Arctic mutation No severe behavioral phenotypes, a feature of preclinical AD No NFTs or neuronal loss, a feature of preclinical AD Genomic homozygosity in order to accelerate pathology and to remove murine endogenous Aβ (heterozygous mice accumulate Aβ, but take longer than homozygous mice.) Overproduction of CTFβ	Analysis of Aβ production, deposition, and Aβ‐associated neuroinflammation Analysis of molecular pathways Analysis of neural network Omics analysis Reverse genetic analysis using the knockout and knock‐in mice Drug development (preventive) Additional gene manipulations (gene editing) Analysis of transcription and splicing of APP Identification of CSF and plasma biomarkers
	NL‐G‐F	C57BL/6J	endogenous APP	APP^KM670/671NL,E693G,I716F^			

A side‐by‐side comparison of key factors to consider in selecting an APP mouse model for preclinical studies. Researchers should decide on a model depending on the specific scientific or therapeutic goal.

APP‐Tg mice recapitulate only a part of AD pathology, and efforts were made to combine them with other mutant mice to further reconstitute the remaining pathological hallmarks. PS1 is a constituent of the γ‐secretase complex that cleaves the C terminal fragment of APP generated by β‐secretase (CTF‐β) to produce Aβ (De Strooper *et al*, 1998). PS1 mutations cause the majority of FAD cases (Karch *et al*, 2014). The overexpression of mutant PS1 or a knock‐in pathogenic *PSEN1* gene mutation alone did not induce Aβ pathology, presumably because the absolute amount of pathogenic longer Aβ such as Aβ_42_ and Aβ_43_ generated from mouse APP was insufficient (De Strooper *et al*, 1995). Alternately, mouse Aβ might have low amyloidogenic potential that might be caused by the existence of three different amino acids compared to human Aβ (Chui *et al*, 1999; Guo *et al*, 1999; Schmitz *et al*, 2004; Xu *et al*, 2015). However, the combination of these mice with human APP‐overexpressing Tg mice increased pathogenic Aβ production and conferred amyloidogenicity, which resulted in accelerated Aβ deposition, behavioral deficits, and neuronal loss. These combinations include Tg2576 and PS1_M146L_ Tg (Holcomb *et al*, 1998), APP_KM670/671NL_ Tg and PS_A246E_ Tg (Borchelt *et al*, 1996, 1997), APP751_KM670/671NL‐V717I_ Tg and PS_M146L_ Tg (Schmitz *et al*, 2004), and APP _KM670/671NL‐V717I_ and *PSEN1* ^*M233T/L235P*^ knock‐in (Casas *et al*, 2004). Oakley *et al* (2006) generated 5XFAD mice carrying five FAD mutations in APP and PS1 transgenes (APP_K670N/M671L/I716V/V717I_ Tg and PSEN1_M146L/L286V_ Tg) driven by the Thy‐1 promoter. These mice exhibited cerebral Aβ pathology and gliosis as early as 2 months of age, synaptic degeneration and neuronal loss, and developed progressive cognitive deficits as early as 4–5 months. However, the 5XFAD mice also failed to develop NFTs despite their aggressive phenotypes and pathological changes (refer to section “Limitations of first‐generation mouse models”).

In efforts to replicate NFT pathology, crossbreeding of mutant Tau‐Tg mice with APP‐Tg mice enhanced tau pathology in the limbic system and olfactory cortex without affecting Aβ pathology (Tg2576 and JNPL3: Lewis *et al*, 2001; APP23 and JNPL3: Bolmont *et al*, 2007). Oddo *et al* (2003) generated a triple Tg model, 3xTg‐AD mice, which overexpress APP_swe_, and Tau_P301L_ transgenes on a PS1_M146V_ knock‐in background. The mice exhibit neuropathology similar to AD patients, including the formation of Aβ plaques and NFTs, together with gliosis, synaptic damage, and memory deficits. However, the introduced mutations in the *Mapt* gene that encode tau protein are not causes of AD but rather of frontotemporal dementia with parkinsonism linked to chromosome 17 (FTDP‐17). In addition, the overexpression of multiple genes causes an increased risk of artificial phenomena making it difficult to interpret the results. APP‐Tg mice crossbred with *Mapt* knockout mice exhibited improvements in memory deficits and survival in APP‐Tg mice, which suggests that tau may possibly confer Aβ toxicity (J20: Roberson *et al*, 2007; APP23: Ittner *et al*, 2010). Several combinations, such as APP‐Tg mice crossbred with BACE1 knockout mice (Ohno *et al*, 2004) or with apolipoprotein E4 (ApoE4) knock‐in mice (Fryer *et al*, 2005), may be more useful for specific applications.

## Studies on first‐generation mouse models

APP‐ and APP/PS‐overexpressing mice exhibit key features of amyloid pathology that have allowed them to be applied in AD research. Although specific details of amyloid pathology such as plaque age of onset, size and regional distribution, and Aβ species content vary depending on the line, APP‐overexpressing mice recapitulate aspects of cerebral Aβ accumulation, including production and deposition of Aβ and associated neuroinflammation (microgliosis and astrogliosis). In some cases, downstream pathologic consequences of Aβ deposition in overexpressing mice, such as tau hyperphosphorylation, formation of dystrophic neurites, loss of synaptic markers, and the accumulation of BACE1 (Zhao *et al*, 2007), appear similar to those observed in AD. Other effects of Aβ deposition in overexpressing mice may also be relevant to AD. For example, 5XFAD mice exhibit neuron loss and memory deficits that are associated with amyloid pathology (Oakley *et al*, 2006). Importantly, BACE1 knockout abolishes Aβ deposition in 5XFAD mice and at the same time prevents both memory deficits and neuron loss in this and other mouse models (Ohno *et al*, 2007). Thus, cerebral Aβ accumulation is responsible for neuron loss and memory deficits in these lines, rather than transgene overexpression, although effects of β‐CTF overexpression cannot be ruled out.

APP‐overexpressing mice have also been useful in validating and assessing BACE1 and γ‐secretase inhibition as a therapeutic strategy for AD. BACE1 gene knockout abrogates cerebral Aβ accumulation in all APP‐ and APP/PS‐overexpressing mice tested to date (Luo *et al*, 2001, 2003; Ohno *et al*, 2004, 2007; Laird *et al*, 2005; McConlogue *et al*, 2007; Rabe *et al*, 2011), validating BACE1 as the major β‐secretase enzyme in the brain. Subsequently, overexpressing mice were used to screen small molecule inhibitors of BACE1, some of which could reduce Aβ levels in the brain and CSF by 90% or more. Some of which have advanced to clinical trials where they show similar Aβ lowering effects in human CSF (May *et al*, 2015; Neumann *et al*, 2015; Kennedy *et al*, 2016; Cebers *et al*, 2017). Another application of overexpressing mice translated successfully to humans is the preclinical testing of the anti‐Aβ antibody aducanumab. Plaque‐bearing Tg2576 mice that were chronically treated with aducanumab experienced a dose‐dependent reduction of cerebral Aβ levels by up to ~70% compared to vehicle (Sevigny *et al*, 2016). The mechanism of aducanumab‐mediated Aβ reduction appeared to involve binding to Aβ deposits in both human AD and aged Tg2576 mouse brains stimulating microglial phagocytosis of Aβ.

Criticisms of first‐generation mouse models have often focused on the failure of γ‐secretase‐based medications. However, mouse work (De Strooper *et al*, 1999; and many follow up studies in overexpressing models) had predicted almost all the side effects seen in human trials, long before phase III clinical trials were halted. Further work into the potential of tackling γ‐secretase in a safe manner is based on overexpressing mice (Weggen *et al*, 2001) and novel insights into more safe approaches continue (De Strooper, 2014), illustrating the utility of the overexpression paradigm for certain types of preclinical studies specifically targeting Aβ production and Aβ deposits, and possibly also for certain pathophysiologies associated with amyloid plaques such as neuroinflammation.

## Limitations of first‐generation mouse models

**Figure 3:**
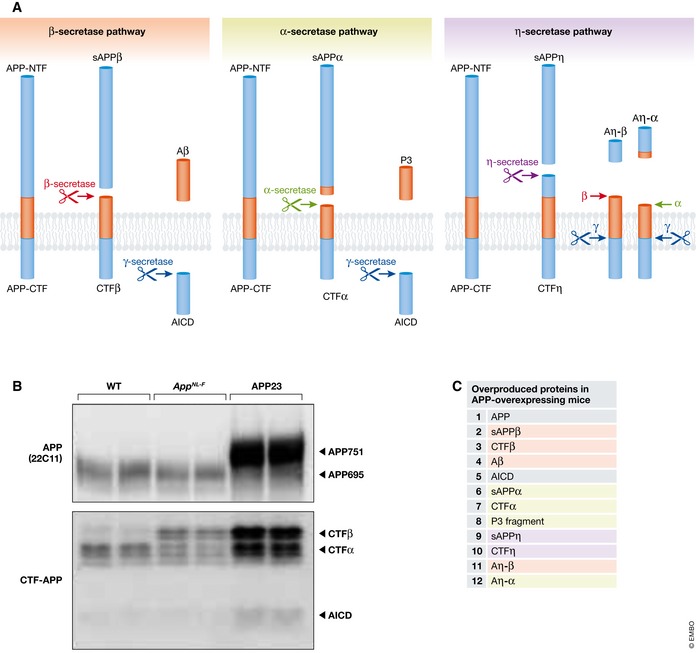
Proteolytic processing of APP in wild‐type and Tg mice (A) APP processing by β‐secretase, α‐secretase, and η‐secretase pathways, respectively. (B) Western blot analysis of APP and APP‐derived fragments in wild‐type (WT), *App* ^*NL‐F*^ *,* and APP23 mice indicated that only APP23 mice produced APP and non‐Aβ APP fragments in substantial abundance. *App* ^*NL‐F*^ mice overproduce CTFβ; however, *App* ^*NL*^ mice produce the same amount without Aβ deposition (Saito *et al*, 2014), therefore serving as relevant negative controls. (C) Proteins that are overproduced in APP‐overexpressing mice.

APP undergoes sequential limited proteolysis catalyzed by proteases, collectively termed “secretases” (Fig 3A). In first‐generation transgenic mouse models, APP overexpression therefore results in the overproduction of various APP fragments in addition to Aβ (Fig 3). This makes it technically difficult to distinguish between the functional effects of additional Aβ and of other overproduced fragments. It is reasonable to assume that some of the phenotypes of the double and triple transgenic mutant mice might be of uncertain relevance to Alzheimer's disease. Box 1 summarizes the mutant APP or APP/PS‐overexpressing mouse models, including prospective ideas that may be experimentally validated to better consolidate the APP overexpression paradigm with human AD clinical pathogenesis (Huang & Mucke, 2012; Saito *et al*, 2014; Palop & Mucke, 2016).

**Box 1: Limitations of mutant APP‐ and APP/PS‐overexpressing mouse models**

- Transgene insertion may destroy endogenous gene loci (Kuro‐o *et al*, 1997; Verret *et al*, 2012; Saito *et al*, 2016).
- Absence of non‐coding regions of the *App* gene precludes the analysis of splicing of APP mRNA and transcriptional regulation involving these gene regions (Nicolas *et al*, 2016).
- Non‐matched negative controls due to variable transgene copy number and insertion site.
- Overexpressed APP interacts unphysiologically with cellular proteins such as kinesin via JIP‐1 (Gunawardena & Goldstein, 2001; Chiba *et al*, 2014; Cassar & Kretzschmar, 2016; Laßek *et al*, 2016).
- Overproduced non‐Aβ APP fragments may interact unphysiologically with cellular proteins (Chang & Suh, 2005; Mitani *et al*, 2012; Nicolas & Hassan, 2014; Kerridge *et al*, 2015; Nhan *et al*, 2015; Willem *et al*, 2015; Xia *et al*, 2016). See Fig 3.
- Non‐specific ER stress may arise in APP/PS‐overexpressing mice (Barbero‐Camps *et al*, 2014; Chaudhari *et al*, 2014; Reinhardt *et al*, 2014; Borkham‐Kamphorst *et al*, 2016; Saito *et al*, 2016).
- Aβ species may appear that are different from those found in clinical AD brain (Saido *et al*, 1995; Saito *et al*, 2016).
- Atypical region specificity of Aβ pathology arises. Different Tg mice use different promoters to drive APP transgene expression that may affect Aβ in vivo propagation (Jucker & Walker, 2013). Höfling *et al* (2016) showed differences in expression level and brain regional patterning of exogenous APP among different APP‐Tg mouse lines.
- Inconsistent drug effects occurred in some cases (Duggan & McCarthy, 2016; Ohno, 2016).
- Crossbreeding with other mutant mice can generate additional artificial phenotype(s) (Higuchi *et al*, 2012; Saito *et al*, 2014). Refer to section “Limitations of first‐generation mouse models” for details.
- Crossbreeding with particular mouse strains increase premature death in some lines (Tg2576 and TgCRND8; Carlson *et al*, 1997; Chishti *et al*, 2001).

In the absence of further validation in patient material or additional animal models in most cases, concerns remain with transgenic overexpression. APP overexpression for instance might induce behavioral abnormalities prior to Aβ pathology (Hsiao *et al*, 1996; Mucke *et al*, 2000), as Aβ pathology arises much earlier than overt disease onset in humans. Apart from potential overexpression artifacts, it has been difficult to standardize the phenotypes of the various models because of their construction with different promoters, transgene constructs, and mouse strains (Webster *et al*, 2014; Foley *et al*, 2015). In summary, further work is needed to reevaluate published results and to be aware that some phenotypes might be the result of APP‐ or APP/PS‐overexpression and not part of normal disease pathology. For instance, the extremely early lethality of calpastatin (*CAST*)‐deficient APP23 mice, half of which die within 10 weeks (Higuchi *et al*, 2012; Saito *et al*, 2014), clearly contradicts the chronic progressive nature of AD. Notably, intrinsic mouse–human differences cannot be ruled out as contributing factors as well (Espuny‐Camacho *et al*, 2017).

## Second‐generation mouse models

To overcome intrinsic drawbacks of the APP overexpression paradigm, mouse models utilizing an *App* knock‐in strategy were generated to overproduce pathogenic Aβ such as Aβ_42_ without overexpressing APP. Single *App* knock‐in mouse models were generated in which the murine Aβ sequence was humanized by changing three amino acids that differ between mice and humans (G676R, F681Y, and H684R) and introduced two FAD mutations (KM670/671NL: Swedish and I716F: Beyreuther/Iberian mutations) into the endogenous mouse *App* gene (Saito *et al*, 2014). The identification of the Beyreuther/Iberian mutation using a phenylalanine scan (Lichtenthaler *et al*, 1999) opened up the possibility for a knock‐in strategy because this particular mutation increased the Aβ_42_/Aβ_40_ ratio by a factor of 30 *in vitro*. This mutation was subsequently identified as a cause for an aggressive form of FAD in Iberia (Guerreiro *et al*, 2010).

Mice that carry NL‐F mutations, denoted *App* ^*NL‐F*^, exhibited increased Aβ_42_ production and a high Aβ_42_/Aβ_40_ ratio without alterations in the expression levels of APP or other fragments. The exception was that *App* knock‐in mice produced more CTF‐β and thus _s_APP_β_ compared with wild‐type mice because of the Swedish mutation (Saito *et al*, 2014). Due to the increase in CTF‐β and concomitant decrease of CTF‐α in the *App* knock‐in mice, the total amount of CTF in *App* knock‐in mice remains the same as in wild‐type mice. To examine the effect of increased CTF‐β and _s_APP_β_ in this case, *App* ^*NL*^ mice were generated that carried only the Swedish mutation and we confirmed that this amount of CTF‐β and _s_APP_β_ exert no effects on the pathology or cognitive function of the mice (Saito *et al*, 2014; Masuda *et al*, 2016). The high levels of Aβ_42_ in *App* ^*NL‐F*^ mice led to pathological Aβ deposition in the cerebral cortex and hippocampus, which was accompanied by enhanced neuroinflammation, that is, infiltration of astrocytes and microglia that surround plaques from 6 months of age. Of particular note, the amyloid plaques in *App* ^*NL‐F*^ mice mainly consisted of pathogenic Aβ_1/3pE‐42_ (Saido *et al*, 1995) in a manner similar to the brains of AD patients, whereas the amyloid plaques in APP‐Tg mice were predominately composed of Aβ_1–40_ and were unphysiologically large, compared to those observed in *App* knock‐in mice and AD patients. A notable exception is the 5XFAD mice, which have amyloid deposits with an over twofold greater amount of Aβ_1–42_ as compared to Aβ_1–40_ (Oakley *et al*, 2006). Synaptic alterations in *App* ^*NL‐F*^ mice were also identified by the loss of presynaptic synaptophysin and postsynaptic PSD95 (Saito *et al*, 2014).

*App* ^*NL‐F*^ mice developed memory dysfunction at 18 months of age as detected by the Y‐maze test. In addition, Masuda *et al* (2016) analyzed the knock‐in mice using IntelliCage and determined that the *App* ^*NL‐F*^ mice exhibited various cognitive dysfunctions, including deficits in spatial memory and flexible learning, enhanced compulsive behavior, and reduced attention performance, depending on the age and pathology of the mice (Masuda *et al*, 2016). *App* knock‐in mice that harbor a third mutation, an E693G Arctic mutation (*App* ^*NL‐G‐F*^), were also generated that makes Aβ more oligomerization/fibrillization‐prone (Cheng *et al*, 2007; Gessel *et al*, 2012), and these mice exhibited threefold faster and greater AD pathology and cognitive abnormalities compared with *App* ^*NL‐F*^ mice.

Reaume *et al* (1996) generated *App* knock‐in mice that harbored the Swedish mutation (K670N/M671L) with humanization of the murine Aβ sequences (*App* ^*NLh/NLh*^). These mice overproduced human Aβ_40_ and Aβ_42_ without overexpressing APP; however, they failed to deposit Aβ in the brain at up to 22 months of age. This group subsequently crossbred their *App* knock‐in mice with mutant *PSEN1* knock‐in mice (Flood *et al*, 2002; Malthankar‐Phatak *et al*, 2012), and the double knock‐in mice successfully exhibited Aβ pathology without depending on the overexpression paradigm. The double knock‐in mice, *App* ^*NLh/NLh*^ × *PSEN* ^*P264L*^, exhibited less aggressive pathology compared with the double transgenic mice, likely because of the lower expression levels of APP and PS1. These mice are likely more difficult to use because of their double homozygous nature but have, for reasons that are unclear, not been used extensively by the community yet bear reassessment as experimental tools.

Li *et al* (2014) produced *App* knock‐in mouse models using multiple pathogenic mutations. The mice carried the Swedish (K670N/M671L), Dutch (E693Q), and London (V717I) mutations with the Aβ sequence humanized. The Dutch mutation causes intensive cerebral amyloid angiopathy (CAA) in humans, which results in brain hemorrhage and early mortality (Levy *et al*, 1990; Van Broeckhoven *et al*, 1990). Thus, this mutation is not a cause of FAD; however, its discovery inspired the first identification of an FAD mutation in the *App* gene (Goate *et al*, 1991; Hardy, 2017). These mice alone developed minimal Aβ deposits throughout life until the authors crossbred them with *PSEN1* ^*M146V*^ knock‐in mice. The double knock‐in mice exhibited an age‐dependent deposition of Aβ not only in the parenchyma of the cerebral cortex but also the cerebral vasculature in a manner similar to human CAA pathology. Consistently, the double knock‐in mice without the Dutch mutation exhibited virtually no vascular pathology. They likely would not have had to introduce the *PSEN* knock‐in mice if they had used the Beyreuther/Iberian mutation instead of the London mutation in the mouse *App* gene. Nevertheless, the Dutch mutation‐harboring knock‐in mice can be considered to represent relevant models for CAA.

## Studies on second‐generation mouse models

New studies suggest that a re‐examination of previous results obtained using first‐generation mouse models with their second‐generation counterparts is good practice. We previously reported that the activation of calpain, a calcium‐activated cysteine protease, is associated with Aβ plaque formation in the brains of AD patients and APP23 mice (Higuchi *et al*, 2012). Genetic ablation of calpastatin (*CAST*), a calpain‐specific inhibitor protein, exacerbated amyloid deposition, neuroinflammation, tau phosphorylation, and somato‐dendritic atrophy. Notably, when APP transgenic mice were crossed with *CAST* knockout mice, there was increased mortality (Higuchi *et al*, 2012) where half of the mice died in 10 weeks for unknown reason(s). In contrast, the double mutant *App* ^*NL‐F*^ mice crossbred with *CAST* knockout mice lived as long as wild‐type mice, indicating that the early lethality demonstrated in APP‐Tg crossbred with *CAST* knockout mice was inconsistent with the chronic nature of AD (Saito *et al*, 2014). Furthermore, Aβ was suggested to induce calpain‐dependent conversion of p35 to p25, a CDK5 activator, which may play an important role in AD pathogenesis (Seo *et al*, 2014). *App* ^*NL‐F*^ mice crossbred with *CAST* knockout mice, in which calpain is hyper activated, did not exhibit conversion of p35 to p25 despite the finding that calpastatin deficiency increases Aβ amyloidosis in the crossed mice (Saito *et al*, 2016). Thus, the conclusion that Aβ accumulation can cause p25 generation in neurons reported in 5XFAD mice may need to be revisited, as it might be caused by a non‐specific increase in calcium (Barbero‐Camps *et al*, 2014; Reinhardt *et al*, 2014) that might be unique to APP/PS1 double transgenic mice (Reinhardt *et al*, 2014), although further work is required to draw firm conclusions. A third result where differences between first and second‐generation mouse models are evident involved the reported down‐regulation of Na_v_1.1, a sodium channel expressed in PV‐positive interneurons in the APP‐Tg mouse line J20 and its resultant effect on epilepsy and AD phenotypes (Verret *et al*, 2012). In contrast, in *App* ^*NL‐F*^ mice, *App* ^*NL‐F*^ mice crossbred with *CAST* knockout mice, or APP23, down‐regulation of Na_v_1.1 was not observed (Saito *et al*, 2016) although it is possible that *CAST* deficiency makes calpain hyper activated (Higuchi *et al*, 2005; Takano *et al*, 2005). Hypofunction of Na_v_1.1 has been also observed in other mouse models such as Tg2576, TgCRND8, and BACE1 transgenic mice (Kim *et al*, 2007; Corbett *et al*, 2013; Hamm *et al*, 2017), and the effect of amyloid on Na_v_1.1 expression and its phenotypic consequences in AD mouse models should continue to be reviewed and validated in future studies.

Several basic findings using the second‐generation mouse models have advanced the basic biology of AD. Hama *et al* (2015) developed a new sorbitol‐based optical clearing method referred to as Sca*l*eS that preserves the cellular structure of the tissue and proteins, including their immunochemical epitopes, enabling a 3D analysis of plaque deposition. *App* ^*NL‐F*^ mice treated with Sca*l*eS allowed quantitative visualization of Aβ in an entire hemisphere, mapping of the 3D network of amyloid plaques in association with the vascular structure, and tracking of single plaques via successive light microscopy (LM) and electron microscopy (EM) observations. 3D images of microglial activation during amyloidosis of *App* ^*NL‐F*^ brains demonstrated that microglia association and active inflammation occur at an early stage of plaque formation. Such clearing methods combined with AD mouse models enable analysis of the degree of Aβ burden in larger brain volumes compared with conventional immunohistochemistry and are also applicable for the verification of immunotherapy (Sevigny *et al*, 2016) by visualizing therapeutic anti‐Aβ antibody binding to regional amyloid *in situ*.

One proposed mechanism for memory loss in AD is the destabilization of mushroom‐shaped postsynaptic spines, which may play an important role in memory storage. In accord, several reports indicate a reduction in mushroom spines in AD brain. In *App* ^*NL‐F*^ mice, Zhang *et al* (2015) demonstrated that hippocampal mushroom spines are lost and the STIM2 (stromal interaction molecule 2)‐nSOC (neuronal store‐operated calcium entry) pathway is altered as early as 3 months of age in a time‐dependent manner. The authors demonstrate the relationship between extracellular Aβ_42_ and spine loss concluding that Aβ_42_‐induced hyperactivation of mGluR5 and the subsequent overload of ER Ca^2+^ signaling likely represent the main cause for mushroom spine loss in *App* knock‐in mice. Moreover, an sSOC‐positive modulator NSN21778 recovered the reduction of mushroom spines and memory deficits via activation of transient receptor potential canonical 6 in *App* knock‐in mice (Zhang *et al*, 2016).

The orphan G protein (heterotrimeric guanine nucleotide‐binding protein)‐coupled receptor (GPCR) GPR3 is reported to regulate γ‐secretase activity and Aβ generation without affecting Notch receptor proteolysis (Thathiah *et al*, 2009). Recently, Huang *et al* (2015) demonstrated that genetic deletion of GPR3 reduced amyloid pathology in the brains of the *App* knock‐in models, as well as the APP‐Tg and APP/PS1‐Tg models. However, a reduction in the Aβ_42_/Aβ_40_ ratio following a genetic deficiency of GPR3 was detectable only in *App* ^*NL‐F/NL‐F*^ mice and not in the transgenic mice. In addition, they demonstrated that both the number and volume of amyloid plaques in *App* ^*NL‐F/NL‐F*^ mice crossbred with *Gpr3* ^−/−^ mice were decreased compared with single *App* ^*NL‐F/NL‐F*^ (*Gpr3* ^*+/+*^) mice using 3D analysis with another clearing technique, CLARITY. These findings demonstrate that second‐generation mouse models can be used to evaluate the effect of new therapeutic targets on Aβ pathology.

BACE1 activity is up‐regulated in AD patients, after Aβ deposition, and in traumatic brain injury (Rossner *et al*, 2006). Kizuka *et al* (2015) hypothesized that bisecting *N*‐acetylglucosamine (GlcNAc) stabilizes BACE1 protein during oxidative stress, which results in an increase in Aβ generation. In a more recent paper, they demonstrated the up‐regulation of BACE1 protein and the level of bisecting GlcNAc in *App* ^*NL‐G‐F/NL‐G‐F*^ mouse brains, which was accompanied by an accumulation of oxidative damage (Kizuka *et al*, 2016).

Recently, the *App* knock‐in mice were used to refute the hypothesis that the new Alzheimer candidate gene PLD3 (Cruchaga *et al*, 2014) was involved in APP processing. Crossing of *Pld3* deficient mice with *App* knock‐in mice demonstrated that there was no modulation of Aβ plaque or APP cleavage in these mice (Fazzari *et al*, 2017)

## Limitations of second‐generation mouse models

Like single APP overexpression mice, the knock‐in mice do not exhibit tau pathology or neurodegeneration. This finding suggests that Aβ pathology may account, at least in part, for the cognitive dysfunction in AD via disturbances in neuronal activities because Zhang *et al* 2015 identified a reduction of mushroom spines, distinguishing spine structure at excitatory synapses, in the early stage in these mice. However, the Aβ‐induced memory failure alone might be insufficient to explain all symptoms of AD patients because tauopathy‐accompanying irreversible neurodegeneration has previously occurred at disease onset even in FAD‐mutation carriers (Bateman *et al*, 2012). Therefore, *App* knock‐in mice should be considered “models of preclinical AD”. A summary of features and limitations in *App* knock‐in mice is shown in Box 2.

**Box 2: Limitations of single *App* knock‐in mouse model and potential solutions**

- All the lines carry Swedish mutations (NL), which may exhibit different sensitivity to β‐secretase inhibitors from the wild‐type lines (KM). This is however easy to fix by converting NL to KM by gene editing, which cannot be applied to the APP‐overexpressing mice.
- The Swedish mutation causes an increase of CTF‐β and concomitant reduction of CTF‐α. Consequently, total CTF levels (CTF‐β + CTF‐α) remain unchanged. If CTF‐β possesses particular biological or pathological functions, this may cause artifacts. Still, the amount of CTF‐β in single *App* knock‐in mice is much smaller than that in APP‐overexpressing mice.
- The knock‐in mice possess two or three independent FAD mutations in the *App* gene. There is no evidence for an interaction between the Swedish mutation and Beyreuther/Iberian mutations, but the Arctic mutation increases CTFs (CTF‐β + CTF‐α) by 50% by unknown mechanisms (T Saito & TC Saido, unpublished; Cheng *et al*, 2004) and results in an unnatural Aβ conformation. It is important to perform delipidation pretreatment for the analysis of CTFs (Sato *et al*, 2003; Saito *et al*, 2014).
- The negative control mice, *App* ^*NL*^, accumulate no Aβ throughout life, but appear to induce minor microgliosis specifically in hippocampus and compulsive behavior (Masuda *et al*, 2016).
- The knock‐in mice are used in a homozygous status, in order to achieve early pathology and to remove murine Aβ. It is advised to backcross them with the original line, B6/J, occasionally, to protect the mice from accumulating *de novo* mutations that could cause recessive defects.
- APP may behave different from human APP because the *App* gene except for part of intron 15–17 is a murine sequence. For example, KPI domain‐containing APP variants are not expressed in mouse brain unlike in human brain (Saito *et al*, 2014). Therefore, *App* knock‐in mice may not be suitable for addressing the properties of KPI domain‐containing APP variants in endothelial cells, since these variants are expressed mostly in endothelial cells (Kitazume *et al*, 2010).

The absence of tauopathy and neurodegeneration in these mice, which live less than 3 years, may be simply a matter of AD time course because it requires more than two decades for Aβ amyloidosis to induce cortical tauopathy and neurodegeneration in humans (Bateman *et al*, 2012). To address these questions, further genetic manipulation to study the connection between Aβ pathology and tauopathy/neurodegeneration will be required. There are only three splice variants of tau (*Mapt* gene product) in adult mouse brain whereas there are six in humans. We therefore have generated human tau knock‐in mice, in which all the exons and introns of murine *Mapt* gene have been humanized (Saido *et al*, 2015). The mice are available to the research community from RIKEN. Other genes should not need to be humanized because overexpression of frontotemporal dementia with parkinsonism (FTDP) mutation‐carrying human tau is sufficient to reconstitute tauopathy composed of Neurofibrillary tangles (NFTs) and neurodegeneration (Lewis *et al*, 2000).

Biomarkers for the diagnosis and prognosis of preclinical AD will not only reduce the cost but also shorten the time necessary for drug development. *App* ^*NL‐F*^ and *App* ^*NL‐G‐F*^ mice are the only single knock‐in models that develop Aβ pathology and memory deficits. However, the presence of multiple mutations in the *App* gene, not observed in human patients, could in principle interact with each other in some cases that may not accurately represent clinical AD (Box 2). Thus, *App* ^*NL‐F*^ mice are suitable for analyzing the mechanisms that affect preclinical Aβ deposition compared with *App* ^*NL‐G‐F*^ that may be more useful for analyzing the mechanisms that alter downstream cascades.

Despite these potential drawbacks, *App* knock‐in mice may be useful as preclinical AD models for a number of purposes including (i) identification of biomarker(s) for preclinical AD, (ii) identification of molecules that evoke tauopathy in an Aβ pathology‐dependent manner, (iii) preclinical studies of preventive medicine(s), (iv) a platform for the generation of improved AD model(s) by crossbreeding with appropriate mutant mice (Table 1), and (v) to study the cellular phase of Alzheimer's disease (De Strooper & Karran, 2016), including the progressive response of vascular, astroglia, oligodendrocyte, and microglial cell populations upon amyloid stress. A distinct advantage is that expression from an endogenous promoter ensures that responses are not directed to cells that artifactually overexpress APP.

## Future perspectives on AD mouse modeling

Previous first‐generation transgenic mouse models have made substantial contributions to our understanding of AD pathology. Many of these studies carried out with the best available mouse lines at the time have advanced the understanding of AD. The new second‐generation mouse lines solve some of the previous limitations and point the way to future third generation models. Until such next generation models become available, studies that investigate the interface of results from first‐ and second‐generation models will continue to reveal discrepancies and may in some cases indicate that findings from previous AD models may in part be a consequence of overexpression artifacts. Sorting out the clinically relevant phenotypes and mechanisms will require years of work with all the models but we urge AD researchers to remain vigilant and not to assume textbook status for any previous findings without extensive validation using the most appropriate mouse lines. We further emphasize that preclinical studies, including immunotherapy, may benefit from a re‐examination with new models to identify drug candidates for the preclinical prevention of earlier AD symptoms.

Species differences between rodents and humans in terms of neuroanatomy, genetics, and behaviors are also critical to control (Emes *et al*, 2003; Molnár & Clowry, 2012; Kaas, 2013; Nithianantharajah & Grant, 2013). Key molecules in AD such as Aβ, tau, and ApoE are different between mice and humans in their sequences, pathogenicity or number of isoforms expressed. In addition, immune systems in the brain also differ between mice and humans in certain aspects such as the proportion of microglial phenotypes, or the expression pattern of inflammation‐related genes (reviewed by Franco Bocanegra *et al*, 2017). The development of induced pluripotent stem cells (iPSCs) from AD patients can help to address species differences (Mungenast *et al*, 2016; Sullivan & Young‐Pearse, 2017). Recently, a novel chimeric AD mouse model was developed by transplanting human PSCs into AD mouse brain, showing pathological changes in tau and neurodegeneration in human neurons (Espuny‐Camacho *et al*, 2017). Furthermore, to leverage species differences for near‐clinical studies, we are generating non‐human primate models of AD (Okano *et al*, 2016) with more similarity to humans that could reduce species barriers and limit the time and cost of drug development.

**Figure 4:**
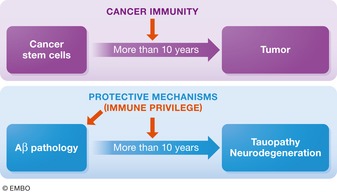
Analogy between cancer immunity and protective mechanism in brain Analogy between cancer and AD. Because of cancer immunity mechanisms, it generally takes cancer stem cells more than a decade to develop pathological cancer. Human brains may possess similar protective mechanisms for AD, which can explain why it takes Aβ amyloidosis decades to induce neurodegeneration, whose identification may improve animal models and protective medications.

AD research can benefit from modern views in the fields of immunology and cancer progression. Cancer stem cells arise in human bodies every day, but fail to develop into cancer in most cases because of cancer immunity (Yarchoan *et al*, 2017; Yeo & Angeli, 2017; Fig 4). We can draw an analogy between cancer immunity and AD protective mechanism(s) in the brain, which can maintain cognition essentially unaffected for more than two decades after the initial deposition of Aβ. If we could identify the molecules responsible for this protection, we should be able to facilitate studies of the major AD pathologies in mice by knocking out or modifying the corresponding genes. In the progressive systemic dysfunction that characterizes the cellular phase of AD, many types of cells are involved with complex feedback and feedforward responses at different disease stages.

The emerging insight that AD is a multidimensional and multicellular process will require more integrated and complex forms of analysis (Tarasoff‐Conway *et al*, 2015; De Strooper & Karran, 2016). Knock‐in animals are most suitable to study these processes with target genes that are expressed in the correct cell types, with appropriate timing and amounts under the control of endogenous promoters. In addition, a number of common variants associated with LOAD have been identified in genes that participate in Aβ clearance or neuroinflammation in genome‐wide association studies (GWAS; Rosenberg *et al*, 2016; Naj *et al*, 2017). Effects of these variants can be precisely assessed by additional gene manipulations in *App* knock‐in mice. Both crossbreeding *App* knock‐in mice with other existing mutants and gene editing, which utilizes novel techniques, such as transcription activator‐like effector nuclease (TALEN) and clustered regularly interspaced short palindromic repeat (CRISPR)/Cas9 will reveal new mechanisms of Aβ pathology (Lee *et al*, 2016).

In conclusion, major steps to improve mouse models for AD are underway. While first‐generation models will remain important and relevant for AD research, the second‐generation mice solve some of the limitations of previous models for both basic and preclinical studies of AD. Standardization and sharing of disease models is essential for the objective interpretation of data, and the second‐generation mice can serve as one of the important baseline models for mechanisms of Aβ pathology obtained in different laboratories or under different conditions. However, every mouse model has limitations, and further side‐by‐side comparisons between *App* knock‐in mice, APP‐overexpressing mice, and other models and observations from human AD patients are required to move toward effective treatments. AD researchers can and should develop an ethic of sharing and comparing data and tools, carefully selecting the most suitable models for their purposes, and analyzing the data with an eye toward maximizing replication. To conquer AD as soon as possible, such collaboration ethics on a global scale will maximize the speed of drug development.
